# Hurst Exponent Analysis of Resting-State fMRI Signal Complexity across the Adult Lifespan

**DOI:** 10.3389/fnins.2018.00034

**Published:** 2018-02-02

**Authors:** Jianxin Dong, Bin Jing, Xiangyu Ma, Han Liu, Xiao Mo, Haiyun Li

**Affiliations:** ^1^School of Biomedical Engineering, Capital Medical University, Beijing, China; ^2^Yanjing Medical College, Capital Medical University, Beijing, China

**Keywords:** hurst exponent, complexity, healthy aging, lifespan, resting-state fMRI

## Abstract

Exploring functional information among various brain regions across time enables understanding of healthy aging process and holds great promise for age-related brain disease diagnosis. This paper proposed a method to explore fractal complexity of the resting-state functional magnetic resonance imaging (rs-fMRI) signal in the human brain across the adult lifespan using Hurst exponent (HE). We took advantage of the examined rs-fMRI data from 116 adults 19 to 85 years of age (44.3 ± 19.4 years, 49 females) from NKI/Rockland sample. Region-wise and voxel-wise analyses were performed to investigate the effects of age, gender, and their interaction on complexity. In region-wise analysis, we found that the healthy aging is accompanied by a loss of complexity in frontal and parietal lobe and increased complexity in insula, limbic, and temporal lobe. Meanwhile, differences in HE between genders were found to be significant in parietal lobe (*p* = 0.04, corrected). However, there was no interaction between gender and age. In voxel-wise analysis, the significant complexity decrease with aging was found in frontal and parietal lobe, and complexity increase was found in insula, limbic lobe, occipital lobe, and temporal lobe with aging. Meanwhile, differences in HE between genders were found to be significant in frontal, parietal, and limbic lobe. Furthermore, we found age and sex interaction in right parahippocampal gyrus (*p* = 0.04, corrected). Our findings reveal HE variations of the rs-fMRI signal across the human adult lifespan and show that HE may serve as a new parameter to assess healthy aging process.

## Introduction

As the elderly population increases, age-related cognitive changes across healthy lifespan emerges as a major concern which can interfere with daily routines and has an impact on quality of life (Hedden and Gabrieli, 2004; St John and Montgomery, 2010; Abrahamson et al., 2012). There is thus a need of more profound comprehension of the law of brain functional changes associated with healthy aging.

Functional magnetic resonance imaging (fMRI) provides non-invasive techniques to explore aging human brain *in vivo* (Bandettini, 2007; Grady, 2008; Dosenbach et al., 2010; Uddin et al., 2010). At present, fMRI study is generally based on task or resting state. Resting state studies of spontaneous fluctuations in fMRI signals have demonstrated huge potential in mapping the brain's intrinsic functional features (Kruger and Glover, 2001; Yan et al., 2009). Ciuciu et al. found that spontaneous brain activities had scale-free dynamics (Ciuciu et al., 2012). He suggested that brain activity observed at rs-fMRI signals exhibits a 1/f-like power spectrum, and the irregular brain activity contributing to this “1/f slope” of the power spectrum was scale-free brain activity (He, 2014). And “scale-free” is the equivalent terminologies for “self-similar” (Expert et al., 2011). Thus, Hurst exponent (HE) has attracted researchers' attention on assessment of spontaneous signal fluctuations in fMRI due to its well displaying the scale-free dynamics by representing the self-similarity of a time series (Maxim et al., 2005; Park et al., 2010). Wink et al. utilized HE to quantify fractal complexity and describe pathological and physiological features, then found that HE increased in bilateral hippocampus with healthy aging (Wink et al., 2006). Liu et al. (2013) suggested that the fractal complexity of resting state BOLD time-series may provide a viable measure to probe the complexity of the underlying brain activity and they found a trend of decreasing complexity of brain endogenous oscillations measured by mean approximate entropy in gray matter with healthy aging. Here complexity can be described as the presence of similar patterns in the rs-fMRI signal. Lipsitz (2004) found that complexity of physiological signals decreased with aging. Further characterizing resting-state brain activity across time with HE analysis could provide new insights into healthy aging process.

In addition, brain healthy aging may differ between genders. Some studies found gender-related differences in prefrontal and limbic regions with emotional and cognitive tasks (Boghi et al., 2006; Hofer et al., 2006; Mcrae et al., 2008; Schulterüther et al., 2008; Keller and Menon, 2009). Ni et.al explored age and gender effects using multifractal analysis of the rs-fMRI series in default mode network (Ni et al., 2014). Lopez-Larson et al. (2011) sought to assess the effects of age and gender, by measuring local brain connectivity of healthy controls using rs-fMRI data. They found that there existed decreased regional homogeneity with aging, and the fastest decline was in the temporal lobe and anterior cingulate. However, their sample size was not large enough to support their findings and using Kendall's coefficient of concordance to compute regional homogeneity values may be sensitive to random noises.

In this study, we proposed a HE based method to detect fractal complexity of the rs-fMRI signal in the human brain across the adult lifespan. Adopting a large sample, we further investigated whether there exist gender difference and interaction between gender and age in HE.

## Materials and methods

### Subjects

Images used in the study are from the Nathan Kline Institute/Rockland Sample (NKI-RS) (Nooner et al., 2012), which is publicly available at the International Neuroimaging Data-sharing Initiative online database. The initial release of the NKI-RS included 207 participants. After excluding subjects with diagnosed mental disorders, who all underwent multimodal brain scans and a battery of psychiatric assessments, 116 healthy subjects with mean age of 44.3 years (age range: 19–85 years, *SD* = 19.4, median = 43, 67 males and 49 females) were selected. Demographic characteristics of the subjects are displayed in Table 1.

**Table 1:** Demographic characteristics of the participants.

**Characteristics**	**Males**	**Females**	**Significance (*p*-values)**
subjects	67 (57.8%)	49 (42.2%)	–
Age, years	42.5 ± 18.0	46.8 ± 21.2	0.25

Participants all went through a scan session utilizing a Siemens Tim Trio 3.0 T 8 channel MRI scanner. All participants were instructed to keep their eyes closed, relax their minds, and not move during the scanning. Each subject completed a 650 s rs-fMRI scan and then a scan session comprised 260 functional volumes. Rs-fMRI scans were collected using an echo-planar imaging sequence [time echo (TE) = 30 ms, time repetition (TR) = 2.5 s, field of view (FOV) = 216 × 216 mm^2^, flip angle (FA) = 80°, matrix size = 64 × 64, number of slices = 38, voxel size = 3.0 × 3.0 × 3.0 mm^3^, 260 volumes]. Each image was viewed to ensure that the whole brain was covered.

### Data preprocessing

All the preprocessing was performed utilizing the Data Processing Assistant for Resting-State fMRI (DPARSF^1^, Yan and Zang, 2010) which is based on Statistical Parametric Mapping (SPM^2^) and Resting-State fMRI Data Analysis Toolkit (REST^3^, Song et al., 2011).

Preprocessing of rs-fMRI images included the following: (i) discarding of the first 10 volumes from each scan for signal equilibration and to make the subjects adapted to the environment, (ii) correcting for temporal shifts in fMRI data acquisition (slice timing correction), (iii) correction for head motion, and participant with head motion >2 degree of rotation, or >2 mm of translation in any direction was excluded, (iv) the Friston-24 model (Friston et al., 1996) was used to regress out head motion effects based on Yan et al.'s (2013) study, and then the signals from white matter and cerebrospinal fluid were regressed out to reduce respiratory and cardiac effects (Fox and Raichle, 2007). Final step is (v) spatially smooth the result data using a 6 mm full width at half maximum (FWHM) Gaussian kernel. Since spontaneous activities may persist in higher frequency bands (Chen and Glover, 2015), temporal filtering was excluded.

### Calculation of HE

The HE is a scalar which measures long-range correlations of a time series. Rescaled Range (R/S) analysis, which is the most common (Hurst, 1951), can effectively examine the temporal complexity of a time series. In this study, we use R/S analysis for HE calculation.

The principle of R/S analysis is demonstrated as follows: given a time series *T* whose length is *L*, and then *T* is divided into *N* intervals and the length of each interval is *A*(1 ≤ *A* ≤ *N*), *A*×*N* = *L*. The *i*-th interval is marked with *I*_*i*_ and the *j*-th element in *I*_*i*_ is marked with *x*_*i, j*_,*j* = 1, 2, 3…*A*, and *e*_*i*_ is the mean value in *I*_*i*_ interval, so

(1) $$\begin{array}{r} y_{i,j}=\overset{j}{\underset{a=1}{\Sigma }}\left(x_{i,a}-e_{i}\right),j=1,2,3\ldots A \end{array}$$

(2) $$\begin{array}{r} R_{i}=\underset{1\leq j\leq A}{\max}\left\{y_{i,j}\right\}-\underset{1\leq j\leq A}{\min}\left\{y_{i,j}\right\} \end{array}$$

(3) $$\begin{array}{r} S_{i}={\left[\frac{1}{A}\overset{A}{\underset{a=1}{\Sigma }}{\left(x_{i,a}-e_{i}\right)}^{2}\right]}^{\frac{1}{2}} \end{array}$$

(4) $$\begin{array}{r} {\left(R/S\right)}_{A}=\frac{1}{N}\overset{A}{\underset{i=1}{\Sigma }}\left(R_{i}/S_{i}\right)=cA^{HE} \end{array}$$

Where c is a constant and HE was defined as the slope of the line fitting the pairs (lnA,ln(RS)A) in a least-square sense. The calculation was implemented by home-made scripts with Matlab 2012 (MathWorks, Natick, MA).

**Figure 1:**
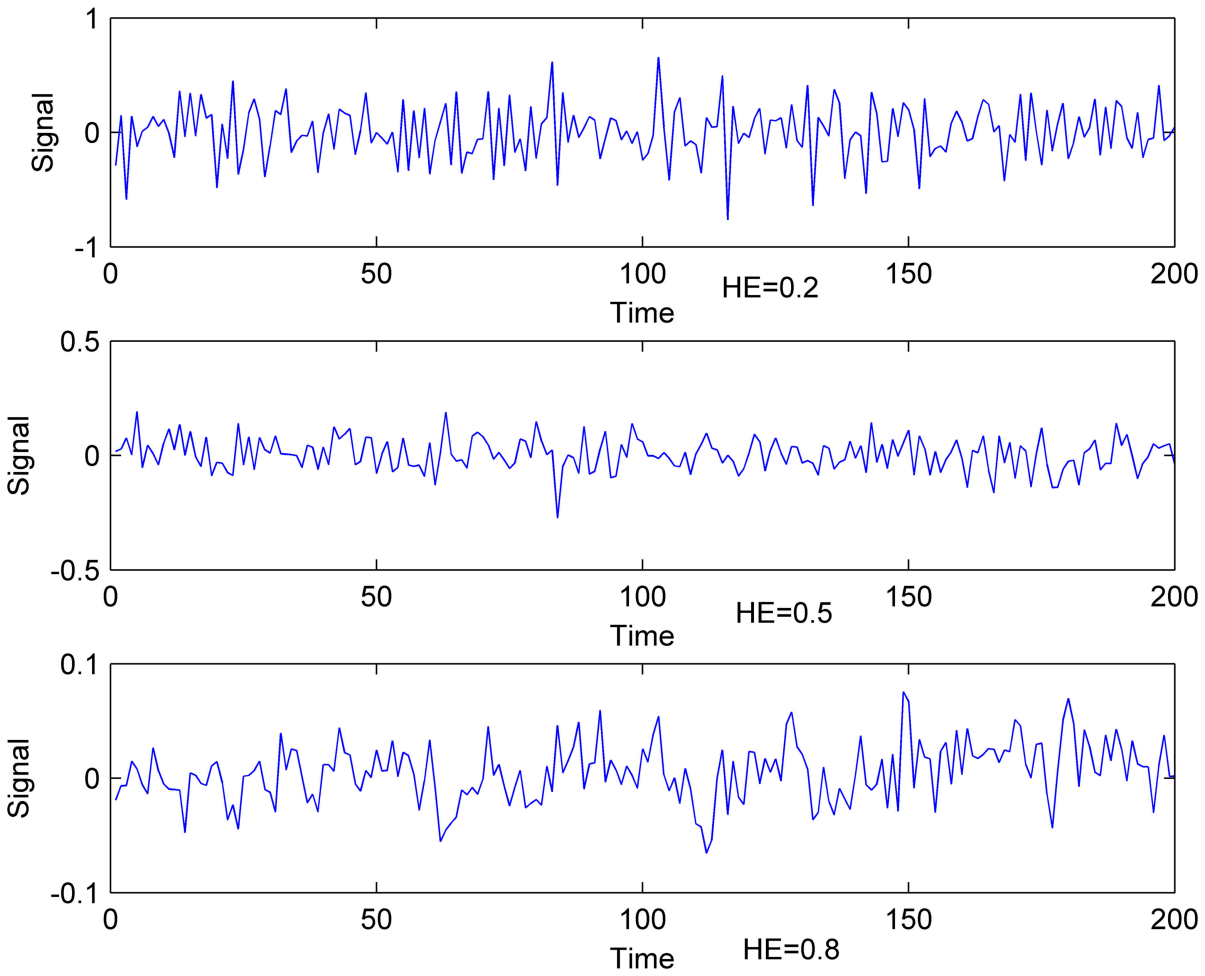
Exemplificative illustrations of time-series with different HE. **Lower**: time- series with HE of 0.8; **Middle**: time-series with HE of 0.5; **Upper**: time-series with HE of 0.2.

HE > 0.5 indicates persistent long memory in the time series, HE < 0.5 implies an anti-correlated time series, and HE = 0.5 reflects a random white-noise time series (Gentili et al., 2015). Therefore, Time series can be divided into three categories (Figure 1). Existed fMRI study (Maxim et al., 2005) showed that HE of voxels in gray matter is about 0.8, and voxels with HE < 0.5 concentrate in cerebrospinal fluid. With the characteristics above, HE is believed to have the capacity to measure brain activity complexity. A higher HE corresponds to a lower fractal dimension, and higher HE means lower complexity accordingly.

### Age effect of HE based on AAL brain atlas

Here we applied Automated Anatomical Labeling (AAL) brain atlas (Tzourio-Mazoyer et al., 2002) to calculate regional HE. This atlas consists of a parcellation of 90 brain regions which is normalized to Montreal Neurological Institute coordinates (MNI coord.) space, and distributed by WFU Pickatlas (Maldjian et al., 2003) software.

The voxel-wise HE were averaged in each of 90 brain regions so that we got the region-wise HE of all the regions for all the subjects. In each brain region, Pearson correlation coefficient was used to measure the correlation between the acquired HE and age. Then, these results were adjusted for multiple comparisons using a False Discovery Rate (FDR) threshold of *q* < 0.05.

### Gender comparison based on AAL brain atlas

The brain cortex was divided into 7 lobar regions including the frontal lobe, insula, limbic lobe, occipital lobe, parietal lobe, sub cortical gray nuclei, and temporal lobe with 90 AAL regions. Values of HE for male and female participants were compared in each lobar region using 2-tailed *t*-test. Then, further assessments were performed on each AAL region within the lobes where there were significant gender differences. These results were adjusted for multiple comparisons using a FDR threshold of *q* < 0.05.

### Age effect of HE based on voxel-wise analyses

Statistics analyses were performed using the REST. Linear age-related effect was estimated by calculating the Pearson correlation coefficient between age and HE within each brain voxels. Significant activations were detected with the cluster size of at lowest 20 voxels, and these results were adjusted for multiple comparisons using a FDR threshold of *q* < 0.05.

### Gender comparison based on voxel-wise analyses

HEs of each of the voxels for males and females were compared using 2-tailed *t*-test. Significant gender differences were detected with the cluster size of at lowest 20 voxels, and these results were adjusted for multiple comparisons using a FDR threshold of *q* < 0.05.

### Interaction between the age and gender

The regions with significant age effect of HE were defined as regions of interest (ROIs), and then interaction between the age and gender was assessed within the ROIs.

## Results

For all age effect analyses, gender was entered as covariate of interest. The mean HE of whole brain gray matter showed significant positive correlation (*r* = 0.35, *p* < 0.01) with age which indicated that complexity of BOLD activity decreased with normal aging.

### Age effect of HE based on AAL brain atlas

**Figure 2:**
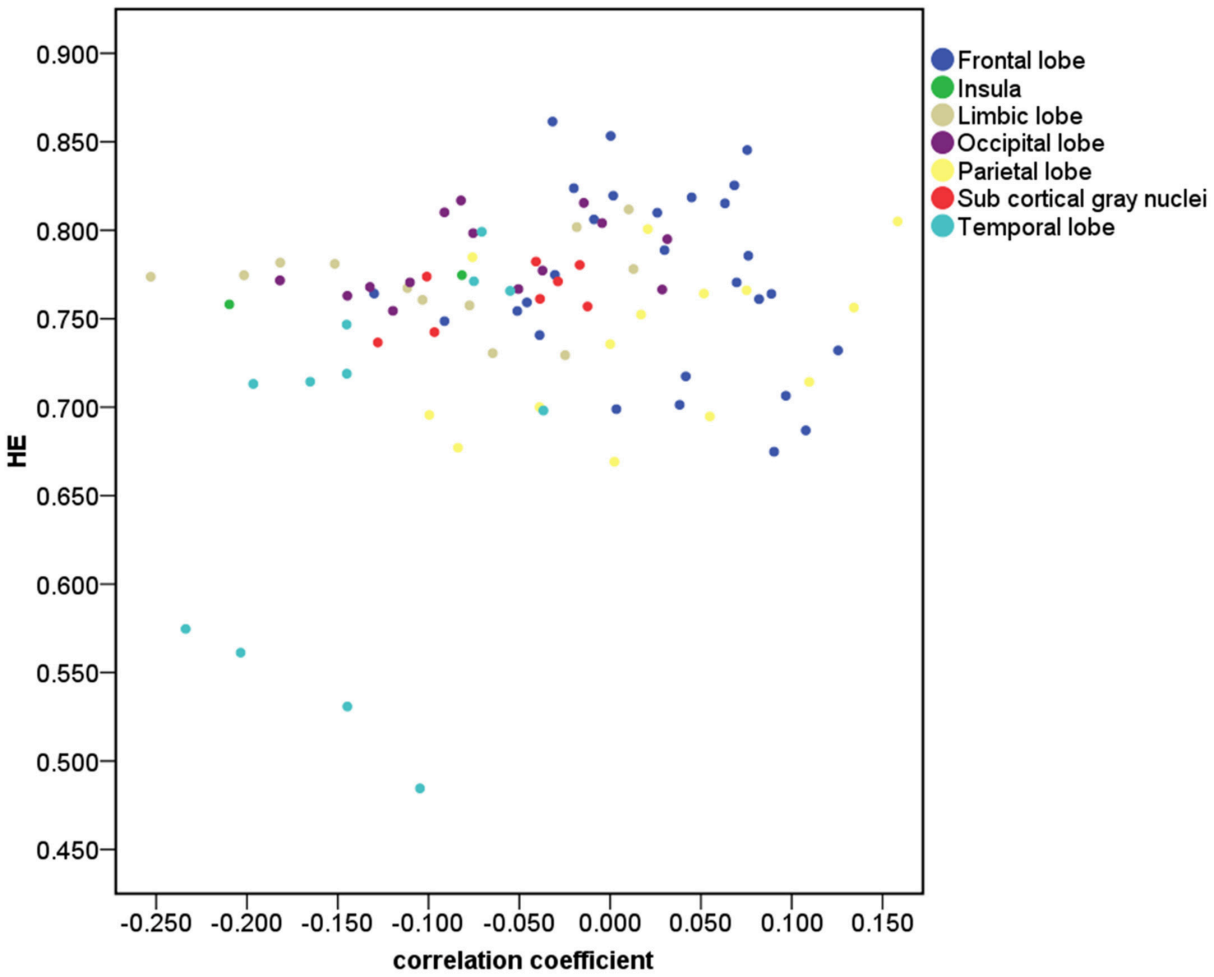
Correlation for the 90 brain regions between HE and age. The brain gray matter was subdivided into 7 lobar regions based on the 90 AAL regions.

Figure 2 depicts that 33 of the 90 brain regions show less complexity with increasing age. Positive age effect was most significant in parietal lobe, including left angular gyrus (*r* = 0.21, *p* = 0.03, corrected) and left superior parietal gyrus (*r* = 0.17, *p* = 0.04, corrected). Significant negative age effect was observed in right insula (*r* = −0.21, *p* = 0.03, corrected), and in limbic lobe including left parahippocampal gyrus (*r* = −0.25, *p* = 0.03, corrected) and right parahippocampal gyrus (*r* = −0.21, *p* = 0.03, corrected). Also, significant negative age effect was observed in temporal lobe, including left superior temporal gyrus (*r* = −0.20, *p* = 0.03, corrected), left superior temporal pole (*r* = −0.20, *p* = 0.03, corrected), and right superior temporal pole (*r* = −0.23, *p* = 0.03, corrected).

**Figure 3:**
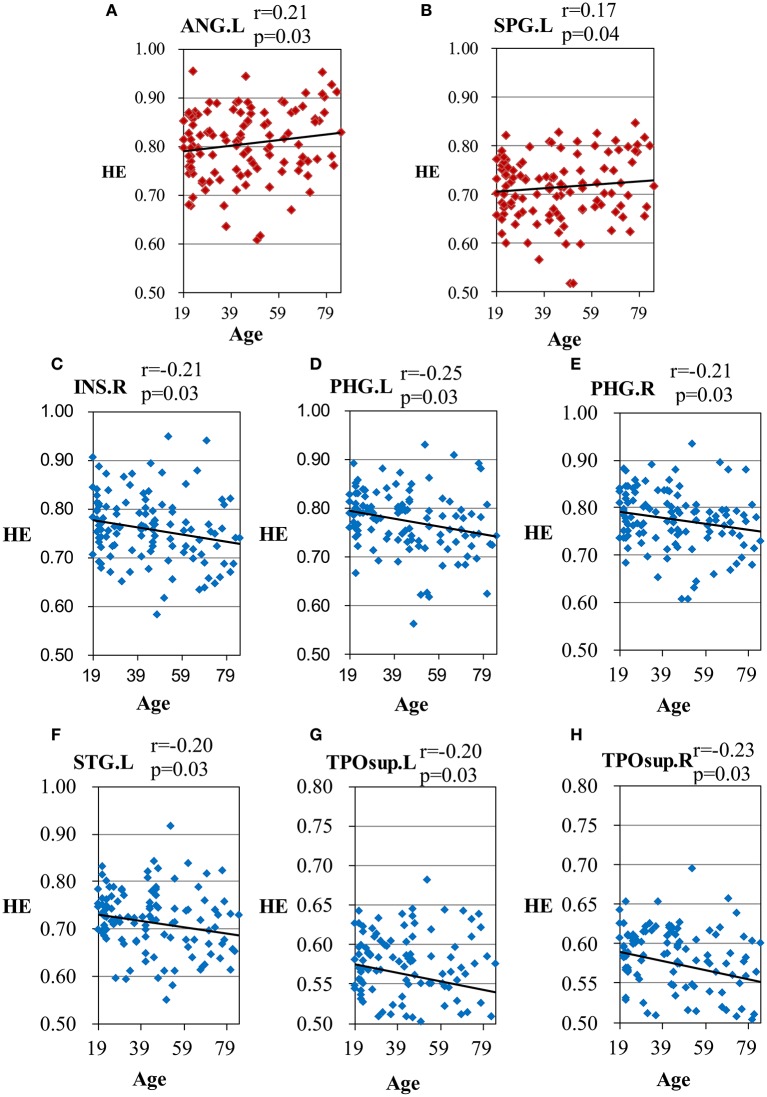
Scatter plots for the significant correlations between age and the HE of the AAL regions. **(A)** ANG.L, left angular gyrus; **(B)** SPG.L, left superior parietal gyrus; **(C)** INS.R, right insula; **(D)** PHG.L, left parahippocampal gyrus; **(E)** PHG.R, right parahippocampal gyrus; **(F)** STG.L, left superior temporal gyrus; **(G)** TPOsup.L, left superior temporal pole; **(H)** TPOsup.R, right superior temporal pole. X-axis, age; Y-axis, the mean HE of the AAL regions. The *p*-values were corrected using the FDR method.

Scatter plots illustrating significant correlations between age and the HE of the AAL regions are shown in Figure 3.

### Gender effect of HE based on AAL brain atlas

**Figure 4:**
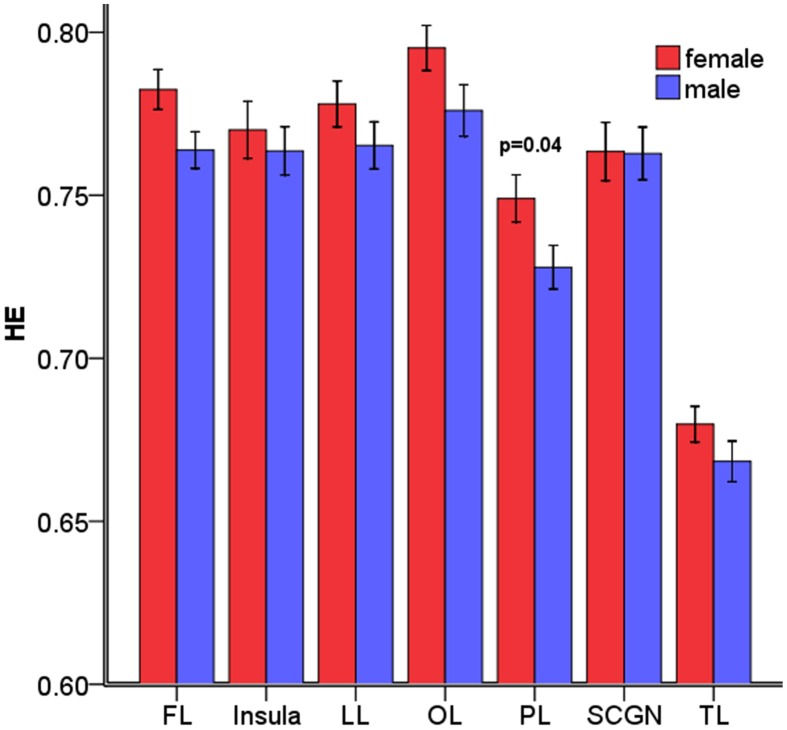
Mean HE of the 7 lobar brain regions between males and females. Significant differences were observed in parietal lobe. Error bar represented standard error. FL, frontal lobe; LL, limbic lobe; OL, occipital lobe; PL, parietal lobe; SCGN, sub cortical gray nuclei; TL, temporal lobe. The *p*-values were corrected using the FDR method.

The overall mean HEs of males and females were significantly different (*p* = 0.04). Then, the differences in HE between genders were further assessed in the 7 lobar brain regions, and we found significant differences between genders in parietal lobe (*p* = 0.04, corrected) where males had smaller HE (see Figure 4).

Furthermore, an examination of the AAL sub regions in parietal lobe was taken. The results showed smaller male HE in the regions listed in Table 2.

**Table 2:** Differences in HE between genders in the AAL sub regions.

**AAL regions**	**Side**	**T**	***p*-values**
Postcentral gyrus	L	2.59	0.04
Postcentral gyrus	R	2.57	0.03
Superior parietal gyrus	R	1.76	0.04
Inferior parietal but supramarginal and angular gyri	R	2.01	0.04
Precuneus	L	1.89	0.04

*L, left; R, right; T, Student's t-test. The p-values were corrected using the FDR method*.

### Age effect of HE based on voxel-wise analysis

**Figure 5:**
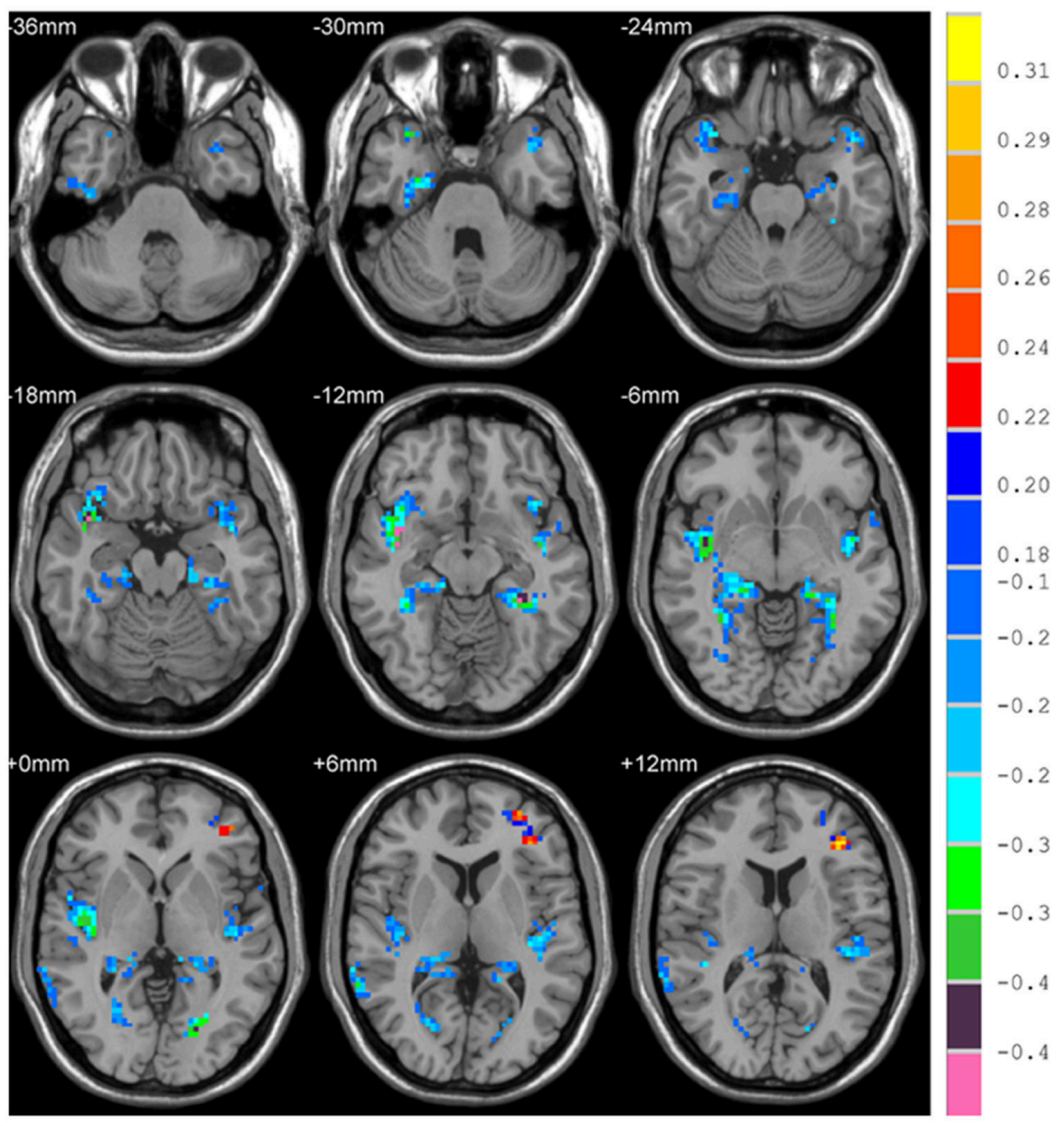
Correlation between HE and age, with positive values showing a decrease of complexity with age.

There is significant positive age effect in frontal and parietal lobe, including left middle frontal gyrus, left triangular part of the inferior frontal gyrus, bilateral superior parietal gyri, bilateral inferior parietal but supramarginal and angular gyri and left angular gyrus. Meanwhile, negative age effects in insula, bilateral parahippocampal gyri, bilateral fusiform gyri, and temporal lobe were found. All results are illustrated in Figure 5 and Table 3, where the positive r values indicate a decrease of complexity with age.

**Table 3:** Correlation between HE and age.

**AAL regions**	**Side**	**Cluster size**	**Peak MNI coord. (mm)**			***r***
			**X**	**Y**	**Z**	
Insula, ParaHippocampal, Fusiform, Temporal_Pole_Sup, Calcarine	R	765	42	9	−18	−0.47
Temporal_Sup, Temporal_Pole_Sup/Mid, Insula	L	267	−42	−9	−9	−0.43
ParaHippocampal, Fusiform, Calcarine	L	304	−30	−39	−12	−0.43
Temporal_Mid/Sup	R	77	66	−45	9	−0.35
Frontal_Inf_Tri, Frontal_Mid/Sup	L	103	−36	36	12	0.33
Parietal_Inf/Sup, Angular	L	128	−36	−54	63	0.31
Parietal_Inf/Sup	R	48	36	−60	60	0.28

*Temporal_Pole_Sup, superior temporal pole; Temporal_Sup, superior temporal gyrus; Temporal_Pole_Mid, middle temporal pole; Temporal_Mid, middle temporal gyrus; Frontal_Inf_Tri, triangular part of the inferior frontal gyrus; Frontal_Mid/Sup, middle/ superior frontal gyrus; Parietal_Inf, inferior parietal but supramarginal and angular gyri; Parietal_Sup, superior parietal gyrus; L, left; R, right; r, Pearson correlation coefficient*.

### Gender effect of HE based on voxel-wise analysis

**Figure 6:**
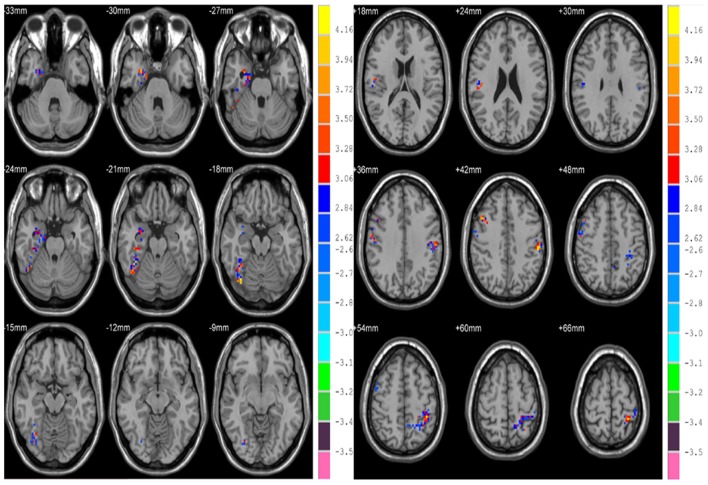
Differences in HE between genders based on voxel-wise analyses.

Differences in HE between genders were assessed in the whole brain gray matter voxels and significant differences were found in voxels of frontal lobe, limbic lobe, occipital lobe and parietal lobe (*p* = 0.01, corrected) (see Figure 6 and Table 4).

**Table 4:** Differences in HE between genders.

**AAL regions**	**Side**	**Cluster size**	**Peak MNI coord. (mm)**			***T***
			**X**	**Y**	**Z**	
ParaHippocampal, Hippocampus	R	79	30	0	−30	4.16
Fusiform, Temporal_Inf	R	77	36	−69	−18	4.34
Frontal_Mid, Postcentral, Precentral	R	108	45	15	42	4.10
Parietal_Inf, SupraMarginal, Postcentral	L	55	−54	−24	42	4.29
Parietal_Sup, Precuneus	L	169	−39	−39	54	4.21

*Temporal_Inf, inferior temporal gyrus; Frontal_Mid, middle frontal gyrus; Parietal_Inf, inferior parietal but supramarginal and angular gyri; Parietal_Sup, superior parietal gyrus; L, left; R, right; T, Student's t-test*.

### Interaction between the age and gender

Only in voxel-wise analysis, a statistically significant interaction between the age and gender was found in right parahippocampal gyrus (*F* = 1.89, *p* = 0.04), but there was no interaction in the other regions.

## Discussion

Rs-fMRI is based on low frequency fluctuations in the BOLD signal, and these fluctuations arise primarily from endogenous oscillations of brain metabolism and neurophysiological activity (Fox and Raichle, 2007; Yan et al., 2009). The complexity of resting-state BOLD signals could provide some evidence of dynamics of intrinsic brain activity (Yang et al., 2013). In this study, we quantified the complexity of rs-fMRI based on HE analysis in a sample of healthy male and female subjects between the ages of 19–85 years old, and found that there existed a significant (*p* < 0.01) positive correlation (*r* = 0.35) between the mean HE of whole brain gray matter and the age of all subjects which means HE increases with age, that is to say, complexity of BOLD activity is reduced with age. Normal aging is accompanied by a loss of complexity in various physiological processes (Lipsitz, 2004; Yang et al., 2013), and aging was found to be associated with significant decrease of complexity in bilateral hippocampus (Wink et al., 2006). Furthermore, aging may facilitate the erosion of both local and long-range connections in the brain, so it could decrease the complexity of spontaneous brain activity (Smith et al., 2014).

In this study, we combined region-wise analysis and voxel-wise analysis to explore complexity of resting-state BOLD signals. The region-wise analysis where the mean of all voxels in one region is given cannot reveal brain regional heterogeneity, and can lead to misinterpretation when opposing effects come out from a single structure. However, the voxel-wise analysis often abide a great many voxels to be computed which provides a lot of false positive results (Lebenberg et al., 2011). Therefore, region-wise analysis and voxel-wise analysis are good complementary tools in our study.

With the region-wise analysis, we found age-related loss of complexity in parietal lobe, specifically the left angular gyrus, and left superior parietal gyrus. With the voxel-wise analysis, on the one hand, we also found this decreased complexity in parietal lobe. On the other hand, we further found age-related loss of complexity in frontal lobe with the voxel-wise analysis, specifically left middle frontal gyrus, and triangular part of the inferior frontal gyrus. With respect to age-related characteristics of complexity of resting-state BOLD signals, some studies used different metrics to investigate characteristics of complexity in brain regions. Liu et al. found decreased complexity in the right middle temporal gyrus, bilateral angular gyri, left middle, and posterior cingulate, left supramarginal gyrus, and left calcarine cortex in aged subjects compared to young subjects (Liu et al., 2013). Compared to Liu et al.'s study, we also found decreased complexity in left angular gyrus. However, we found inconsistent results in right mid temporal gyrus, left mid cingulate, and left calcarine cortex of this study. This may due to the difference of sample size (*n* = 116 in our study vs. *n* = 16 in their study) and age range (19–85 years old vs. two groups, young: age 23 ± 2 and elderly: age 66 ± 3). And what's more, we performed correlation analyses between the HE and age to investigate the age effect on complexity, however, Liu et al. used approximate entropy as a measure of complexity in two groups of healthy subjects consisting of old and young volunteers. Our results about age-related decrease of complexity is most pronounced in parietal and frontal lobe, which is consistent with the regions found by Sokunbi et al.'s study (Sokunbi et al., 2015) where the age range of subjects is similar with ours, and they measured rs-fMRI signal complexity utilizing fuzzy approximate entropy.

Regarding the mechanism for age-related loss of complexity in these brain regions, we know that inferior frontal gyrus is integral for language function and the mirror system (Lai et al., 2010) and important for cognitive control (Schlesinger et al., 2017). It has been suggested that cognitive control is modulated by age (Treitz et al., 2007). Our results about age-related loss of complexity are in agreement with the Lipsitz model which demonstrated healthier system exhibits more complexly in their physiological output and complexity of system decreases with age (Lipsitz, 2004).

On the contrary, we found that age-related increase of complexity is most pronounced in insula, limbic lobe (bilateral parahippocampal gyri), and temporal lobe (left superior temporal gyrus, bilateral superior temporal poles) using two level analyses. The insula plays a vital role in interactions between motor, affective, and cognitive functions (Mathys et al., 2014). We speculate age-related increase of complexity in insula is due to that insula is critical for emotional feeling (Gasquoine, 2014), and with aging, the adult's ability to regulate emotion remains stable and improves in some aspects (Nashiro et al., 2012). Superior temporal gyrus and temporal pole are necessary for understanding perspective taking, movements, and convergence of social knowledge (Lai et al., 2010). In addition, with the voxel-wise analysis, age-related increase of complexity was found in fusiform gyrus and right middle temporal gyrus. Generally, fusiform gyrus is related to cognitive functions (Schenker-Ahmed and Annese, 2013). Complexity increased with age in these regions which suggested that potential compensatory mechanisms may play a role (Sugiura, 2016).

Brain aging may differ between genders. In our study, differences in HE between genders were found to be significant in the parietal lobe (bilateral postcentral gyri and left inferior parietal but supramarginal and angular gyri) on both region-wise and voxel-wise analyses, with females exhibiting higher HE values. In addition, voxel-wise based analysis showed that there were significant differences between genders in the right parahippocampal gyrus and fusiform gyrus. The inferior parietal lobule is a part of attention network, and parahippocampal gyrus is correlated with short-term memory. We conjecture that gender differences occur in the behavioral and cognitive domains because women generally excel in language (Hyde and Linn, 1988), emotional memories (Canli et al., 2002), and facial emotion recognition (Rahman et al., 2004).

In addition, we tested the interaction between the age and gender in terms of HE and found an interaction in right parahippocampal gyrus (*p* = 0.04), suggesting that the age effect on complexity was different between genders in this region.

There are several limitations in our study. We only focused on functional changes without including any structural analysis which could provide a broader view of age-related brain changes by considering structural differences and their associations with functional results. Moreover, cortical atrophy makes spatial normalization difficult for older subjects, and can potentially decrease the signal to noise ratio of the data. Therefore, further investigations are needed to confirm current findings.

## Ethics statement

Data used in preparation of this article were obtained from the International Neuroimaging Data-sharing Initiative (INDI) online database (http://fcon_1000.projects.nitrc.org/indi/pro/nki.html). As such, the investigators within the INDI contributed to the design and implementation of INDI and/or provided data but did not participate in analysis or writing of this report.

## Author contributions

JD made substantial contributions to the conception and design of the work, analysis and interpretation of data for the work, and the draft of the manuscript; BJ and XM made a contribution to the revision of the manuscript; HLiu and XM made a contribution to the conception and design of the work. As the corresponding author, HLi made great contributions to interpretation of data, and determined the final version to be published. All authors have read and approved the final manuscript.

### Conflict of interest statement

The authors declare that the research was conducted in the absence of any commercial or financial relationships that could be construed as a potential conflict of interest.

## Footnotes

^1^http://www.restfmri.net

^2^http://www.fil.ion.ucl.ac.uk/spm

^3^http://www.restfmri.net
